# Responses of Soil Microbial Survival Strategies and Functional Changes to Wet–Dry Cycle Events

**DOI:** 10.3390/microorganisms11112783

**Published:** 2023-11-16

**Authors:** Yaqi Zhang, Chunyi Mo, Yaqing Pan, Pengbin Yang, Xiaodong Ding, Qian Lei, Peng Kang

**Affiliations:** 1 School of Biological Science and Engineering, North Minzu University, Yinchuan 750021, China; zhangyaqi1702@163.com (Y.Z.); mochunyi2023@163.com (C.M.); yangpengbin1022@126.com (P.Y.); dingxd@nmu.edu.cn (X.D.); 2 Shapotou Desert Research and Experiment Station, Northwest Institute of Eco–Environment and Resources, Chinese Academy of Sciences, Lanzhou 730000, China; yaqing_pan@163.com

**Keywords:** wet–dry events, r/K–strategist bacteria, network, microbial community function

## Abstract

Soil microbial taxa have different functional ecological characteristics that influence the direction and intensity of plant–soil feedback responses to changes in the soil environment. However, the responses of soil microbial survival strategies to wet and dry events are poorly understood. In this study, soil physicochemical properties, enzyme activity, and high–throughput sequencing results were comprehensively anal0079zed in the irrigated cropland ecological zone of the northern plains of the Yellow River floodplain of China, where *Oryza sativa* was grown for a long period of time, converted to *Zea mays* after a year, and then *Glycine max* was planted. The results showed that different plant cultivations in a paddy–dryland rotation system affected soil physicochemical properties and enzyme activity, and *G. max* field cultivation resulted in higher total carbon, total nitrogen, soil total organic carbon, and available nitrogen content while significantly increasing α–glucosidase, β–glucosidase, and alkaline phosphatase activities in the soil. In addition, crop rotation altered the r/K–strategist bacteria, and the soil environment was the main factor affecting the community structure of r/K–strategist bacteria. The co–occurrence network revealed the inter–relationship between r/K–strategist bacteria and fungi, and with the succession of land rotation, the *G. max* sample plot exhibited more stable network relationships. Random forest analysis further indicated the importance of soil electrical conductivity, total carbon, total nitrogen, soil total organic carbon, available nitrogen, and α–glucosidase in the composition of soil microbial communities under wet–dry events and revealed significant correlations with r/K–strategist bacteria. Based on the functional predictions of microorganisms, wet–dry conversion altered the functions of bacteria and fungi and led to a more significant correlation between soil nutrient cycling taxa and environmental changes. This study contributes to a deeper understanding of microbial functional groups while helping to further our understanding of the potential functions of soil microbial functional groups in soil ecosystems.

## 1. Introduction

Soil microorganisms play a critical role in regulating soil ecological processes and biogeochemical cycles and participate in and mediate plant–soil feedback (PSF) processes [1,2]. This process is driven by environmental heterogeneity, including the availability of soil nutrients [3], water [4], light [5], and altitude [6]. Floodplain wetland ecosystems are important carbon and nitrogen sinks that play prominent ecological and hydrological roles, providing opportunities for multidimensional studies on PSF responses to temporal, spatial, and even environmental changes [7,8,9]. In particular, the harsh natural conditions (frequent floods and droughts) of the Yellow River floodplain wetlands in northern China make natural and semi–natural ecosystems fragile [10]. They also face pressure from human disturbance. As stated above, the Yellow River enriches agricultural production in Ningxia, and the impact of agricultural activities in this region on the ecosystem also changes the direction or intensity of PSF [11]. The study of soil microbial responses to environmental changes in roaming wetlands has become a topic of interest in climate change research and microbial ecology [12,13].

Meanwhile, changes in the structure and function of soil microbial communities involved in biogeochemical cycles under wet–dry events have received increasing attention [14,15,16]. A recent study indicated that soil microorganisms participate in the decomposition of inert soil organic carbon, which may affect the biogeochemical cycling of key soil nutrient elements under drought conditions [17]. However, wet–dry events in agroecosystems alter soil microbial diversity, community structure, and function [18]. A comprehensive understanding of the soil microbial response patterns under alternating *Zea mays* (dryland) and *Oryza sativa* (wetland) cultivation in the Yellow River floodplain wetlands can help predict agroecosystem responses to current and future environmental changes.

In addition, we were interested in the survival strategies of microorganisms during wet and dry events. Specifically, different microbial taxa exhibit different ecological characteristics [19,20]. For example, r–strategist bacteria are eutrophic microorganisms because of their high growth rate and low resource–use efficiency, and K– and r–strategist bacteria have opposite survival strategies, as K–strategist bacteria are oligotrophic microorganisms [21]. Soil microbial responses to wet–dry events cause differences in the resistance and resilience trade–offs of r– or K–strategists, leading to changes in the microbial community structure [22,23]. In this way, we can also speculate that the soil bacterial r– and K–strategists will also be influenced by wet–dry events.

Although the inter–relationship between spatial variation in environmental factors and PSF is controversial, factors related to climate change (temporal, spatial, and environmental factors) act as drivers that influence soil microbes, thereby influencing soil carbon and nutrient cycling [24,25,26]. In other words, integrating soil nutrient characteristics (soil enzyme activity and soil nutrients) to measure ecosystem functions has become an important way to understand changes in biodiversity and land management [27,28,29]. We conducted this investigation in wetlands (*O. sativa* planting) and drylands (*O. sativa* fields converted to *Z. mays* and *Glycine max* fields for one year) in floodplains in Ningxia through the high–throughput sequencing of soil microorganisms and the determination of soil physicochemical properties. The following questions were addressed: (1) How do soil microbial diversity and r– and K–strategists bacteria respond to plant–soil feedback under wet–dry events? (2) How do soil bacterial and fungal functions respond to wet–dry events?

## 2. Materials and Methods

### 2.1. Study Site and Sample Collection

**Figure 1:**
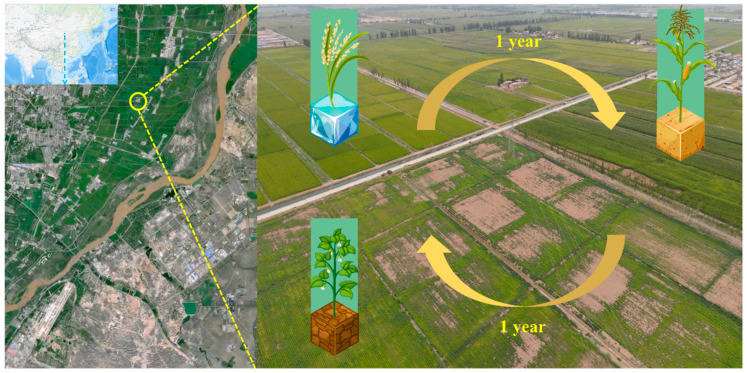
Sampling diagram of the Yellow River beach in northwestern China.

The study site was located in Xingfu Village, Helan County, Ningxia (106°09′–106°30′ E, 35°15′–35°41′ N) and was part of the plain–irrigated cropland ecological zone of the middle and upper reaches of the Yellow River (Figure 1). The figure was obtained from https://apps.nationalmap.gov/viewer/ (accessed on 17 October 2023). In this study, *O. sativa* was grown for eight years, and the soil samples were named *O. sativa*–planting soil (RPS); the *O. sativa* field was then replaced with a *Z. mays* field for one year, and soil samples were named *Z. mays*–planting soil (MPS); then, the *O. sativa* field was changed to a *Z. mays* field for one year and then *G. max* was planted for one year, and soil samples were named *G. max*–planting soil (SPS). Three sample plots (RPS, MPS, and SPS) were divided into nine quadrats, each covering an area of approximately 660 m^2^. Five sampling points were assigned randomly to each quadrat [30]. The litter at the soil–water interface was removed during sampling in the RPS sample plots to avoid plant roots, and 10–20 cm of soil at the soil–water interface was collected and placed in 100 mL sterile centrifuge tubes. The MPS and SPS plots were also sampled at depths of 10–20 cm [16,31]. Plant roots were avoided during sampling, and surface litter was removed before the 2 mm screening. The collected soil samples were immediately placed in a refrigerator at 4 °C and returned to the laboratory.

To reduce the effect of moisture on the soil microbial community structure, we used the method described by Qi et al. (2020) in which MPS and SPS sample soils (50 g) were placed in 200 mL glass bottles, and then sterile water was added to 100% field water capacity (FWC), which was sealed and sterilized together with the RPS sample soil, and cultured in a dark room (20 °C) to stabilize microbial communities [16]. The soil was then air–dried, and subsequent experiments were carried out after the water content was brought to 60% FWC. Some of the soil samples were sent to Novogene Bioinformatics Technology Co., Ltd. (Beijing, China) and stored at a low temperature for the determination of soil bacteria and fungi. The remaining soil was divided into two parts: one for the determination of soil enzyme activity and the other for the determination of soil physical and chemical properties.

### 2.2. Analysis of Soil Physicochemical Properties

The 27 soil samples were sieved and weighed to determine the soil water content (SWC). Briefly, 10 g of soil was weighed in a beaker, 50 mL of deionized water was added, and a conductivity meter (DDS–307A, LEICI Scientific Instruments Co., LTD, Tokyo, Japan) was used to determine the soil electrical conductivity (EC), and a pH meter (PHS–3C, LEICI Scientific Instruments Co., LTD) was used to determine the soil pH [32]. Soil samples were air–dried, and an Elementar vario Macro cube was used to determine the total carbon (TC) and nitrogen (TN) content in the soil. Total phosphorus (TP) was measured using the alkaline potassium persulfate digestion method. The content of total organic carbon in soil (TOC) was determined using the dichromate oxidation method, the content of available nitrogen in soil (AN) was determined using the alkali hydrolysis method, and the content of available phosphorus in soil (AP) was determined using the ammonium molybdate colorimetric method [32].

### 2.3. Determination of Soil Extracellular Enzyme Activities

Fresh soil samples were naturally air–dried, and 1 g of soil samples was weighed, added to toluene extraction, and colorimetrically measured with MUB buffer solution (modified universal buffer solution) to determine the activities of 1,4–α–glucosidase (αG), 1,4–β–glucosidase (βG), 1,4–β–xylosidase (βX), phenol oxidase (POX), β–1,4–Nacetyl–glucosaminidase (NAG), leucine aminopeptidase (LAP), and alkaline phosphatase (SAP) [33,34]. We used the method described by Moorhead et al. (2016) to incorporate αG, βG, βX, and POX into carbon–acquisition enzyme activity [35]; NAG and LAP into nitrogen–acquisition enzyme activity; and SAP into phosphorus–acquisition enzyme activity, which was calculated using the following equation:

Vector Length (Vector L) = SQRT (x^2^ + y^2^) (1)

Vector Angle (Vector A) = DEGREES (ATAN2 (x, y)) (2)

x = ln(αG + βG + βX + POX)/ln[AP], y = ln(NAG + LAP)/ln[AP]. (3)

### 2.4. High–Throughput Sequencing of Soil Bacteria and Fungi

Soil DNA was extracted from the 27 soil samples using the CTAB method. The soil bacterial V3–V4 gene region and fungal ITS1 were amplified using primers (341F and 806R, ITS1F, and ITS2) [36,37]. After PCR amplification, the products were extracted from a 2% agarose gel and purified for quantification. Finally, the amplified products were sequenced on the NovaSeq PE250 platform. Raw sequencing data were stored in the NCBI Sequence Read Archive databases (PRJNA881805 and PRJNA881817).

### 2.5. Data Analysis

QIIME (V1.9.1) software was used to analyze microbiome alpha diversity (OTUs, Shannon, and richness) [38]. The Bray–Curtis distance matrix was used to analyze the beta diversity of the bacterial and fungal communities [39]. Spearman’s analysis was used to analyze the α and β diversity of soil bacteria and fungi and the correlation between soil physicochemical properties and enzyme activities [31]. Then, the “circlize” package in R software (4.1) was used to analyze the level of bacteria and fungi community. Gammaproteobacteria and Bacteroidota were defined as r–strategist bacteria, whereas Alphaproteobacteria and Acidobacteriota were defined as K–strategist bacteria according to the main idea of this study and previous studies [20,40]. The physicochemical properties and enzymatic activities of the 27 soil samples were analyzed using Spearman’s correlation, and the Mantel test was used to describe the correlation [41]. 

To visualize the relationship between r/K–strategist bacteria and functional fungi more intuitively, we calculated the Spearman coefficients for the OTUs of r/K–strategist bacteria and fungi in the three sampling plots (|r| > 0.9, *p* < 0.001) and then established the co–occurrence network with established data (e.g., showing the number of nodes in Gammaproteobacteria, Bacteroidota, Alphaproteobacteria, Acidobacteriota, and fungi) [20]. We further identified environmental factors affecting soil microbial communities and composition through random forest analysis. Subsequently, phylogenetic trees of the r– and K–strategist bacteria were constructed and their relative abundances with major environmental factors were tested using Spearman’s correlation [42]. 

We further analyzed the elemental cycling function of the bacterial communities and predicted it using FAPROTAX [43]. The bacterial functional groups between the two plots were analyzed using one–way and Duncan’s multiple–range tests (*p* < 0.05). The r/K–strategist bacteria with differential bacterial functional groups were correlated and presented using the Cytoscape software (3.7.1) [44]. Furthermore, FUNGuild was also used to predict the ecological functions of fungal communities [45], and Spearman’s correlation analysis was used to further show the relationships between soil physicochemical properties and enzymatic activities with differential bacterial and fungal functions.

## 3. Results

### 3.1. Changes in Soil Physicochemical Properties and Enzyme Activities under Wet–Dry Events

Crop rotation under wet–dry events altered physical and chemical soil properties, and *Z. mays* and *G. max* planting significantly increased soil EC, while TC, TN, TOC, and AN contents were significantly higher in plots after *G. max* planting than in the RPS and MPS sample plots (*p* < 0.05). Soil αG, βG, and NAG contents were higher in the SPS sample plots than in the RPS and MPS sample plots, whereas LAP was significantly lower than RPS and MPS (*p* < 0.05) (Table 1).

### 3.2. Soil Microbial Community Diversity and Composition under Wet–Dry Events

**Figure 2:**
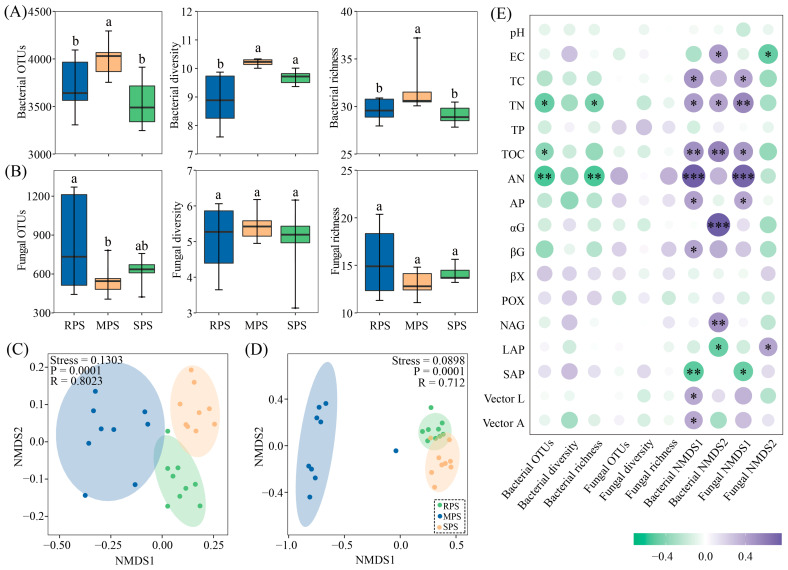
Alpha diversity of soil bacterial (**A**) and fungal (**B**) communities, non–metric multidimensional scale ordering of bacterial (**C**) and fungal (**D**) communities, and Spearman’s correlation with soil physicochemical analysis of RPS, MPS, and SPS plots (**E**) in wet–dry events. RPS: *Oryza sativa*–planting soil; MPS: *Zea mays*–planting soil; SPS: *Glycine max*–planting soil. The differences between different sample plots are shown with lowercase letters (Tukey’s test; *p* < 0.05) (* *p* < 0.05, ** *p* < 0.01, *** *p* < 0.001).

Alpha diversity analysis revealed that *Z. mays* cultivation increased the alpha diversity of the soil bacteria. The wet–dry events had no significant effect on soil fungal diversity but decreased richness (*p* < 0.05) (Figure 2A,B). The NMDS analysis showed that the soil bacterial community composition differed among the three sample plots. Additionally, the fungal community compositions of the RPS, MPS, and SPS sample plots differed (Figure 2C,D). Correlation analysis further indicated that bacterial OTUs and richness were strongly correlated with TN and AN; bacterial NMDS1 and fungal NMDS1 were positively correlated with TC, TN, TOC, AN, and AP and negatively correlated with SAP (Figure 2E). 

### 3.3. Changes in Soil r/K–Strategist Bacteria under Wet–Dry Events 

**Figure 3:**
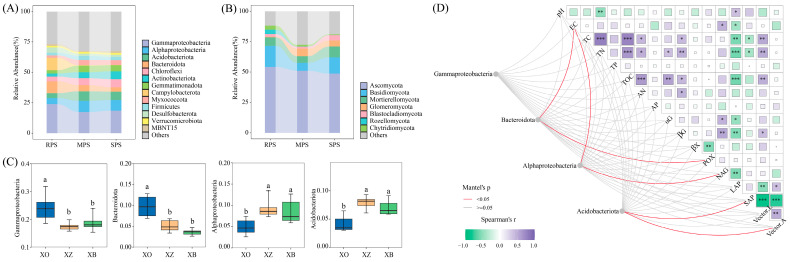
Relative abundance of bacteria (**A**) and fungi (**B**) greater than 1% at the phylum level, the difference in the relative abundance of r– and K–strategist bacteria (**C**), and their correlation with soil physicochemical properties and extracellular enzyme activities (**D**) of RPS, MPS and SPS plots in wet–dry events. The differences between different sample plots are shown with lowercase letters (Tukey’s test; *p* < 0.05) (* *p* < 0.05, ** *p* < 0.01, *** *p* < 0.001).

In addition, the soil bacterial community composition in the three sample plots was dominated by Gammaproteobacteria, Alphaproteobacteria, Acidobacteriota, Bacteroidota, and Chloroflexi. Ascomycota, Basidiomycota, and Mortierellomycota were the dominant phyla in the soil fungal community (Figure 3A,B). We further found that in wet–dry events, the r/K–strategist Gammaproteobacteria, Bacteroidota, Alphaproteobacteria, and Acidobacteriota had significant differences among the three plots (*p* < 0.05) (Figure 3C). The Mantel test showed that Bacteroidota, Alphaproteobacteria, and Acidobacteriota had different correlations with soil physicochemical properties (Figure 3D). 

### 3.4. Relationships between Soil r/K–Strategist Bacteria and Fungi under Wet–Dry Events

**Figure 4:**
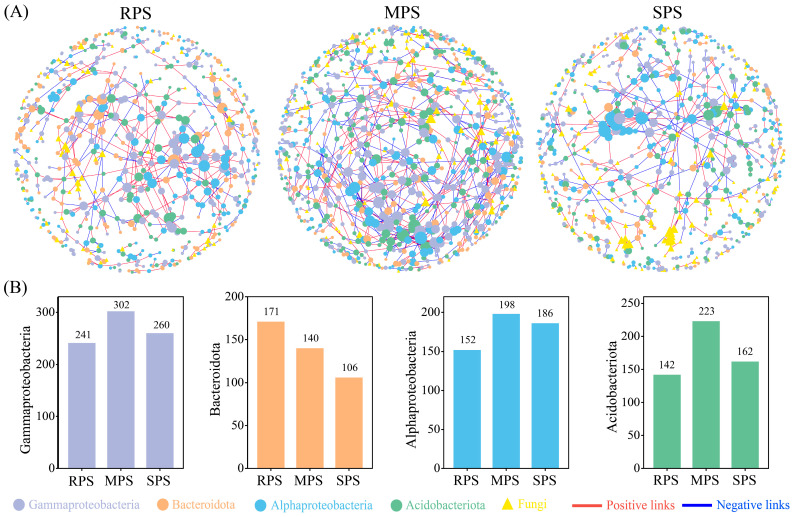
Correlation network analysis (**A**) and number of nodes (**B**) between r– and K–strategist bacteria of RPS, MPS, and SPS plots in wet–dry events. The color of the nodes indicates the different bacterial and fungal phyla, where the circular nodes represent bacteria, and the triangular nodes represent fungi.

Following wet–dry events, MPS sample plots had more complex bacterial–fungal network relationships, where the number of nodes and edges were higher than those of the RPS and SPS sample plots; however, SPS had more stable bacterial–fungal network relationships after *G. max* planting. The ratio of positive–to–negative edges in the SPS plot was higher than those in the RPS and MPS plots, and the number of modules in the SPS plot was also higher. By constructing a functional fungal network, Gammaproteobacteria, Alphaproteobacteria, and Acidobacteriota were found to vary similarly in the bacterial–fungal network (Figure 4A) (Table 2). Notably, MPS had the highest number of nodes (442) for r–strategist bacteria (Gammaproteobacteria and Bacteroidota), followed by RPS (412) and SPS (366). MPS had the highest number of nodes (421) for K–strategist bacteria (Alphaproteobacteria and Acidobacteroita), followed by SPS (348) and RPS (294) (Figure 4B).

### 3.5. Main Environmental Factors Affecting Soil Microbial Community Structure under Wet–Dry Events

**Figure 5:**
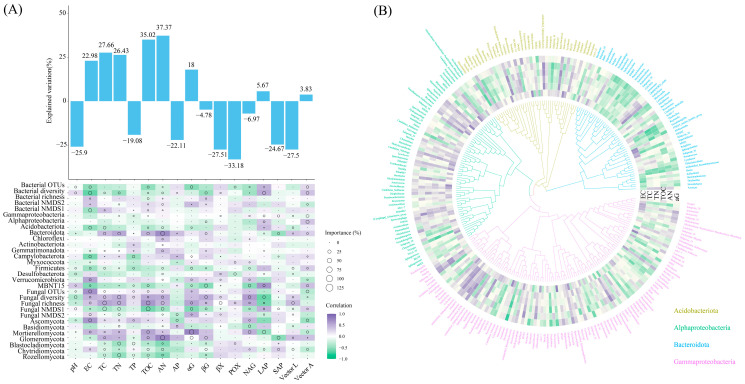
Interpretation of soil environmental factors and extracellular enzyme activities on soil microbial community structure based on correlation and random forest models (**A**), and the phylogenetic distribution of the major influencing factors and r– and K–strategist bacteria (**B**) of RPS, MPS, and SPS plots in wet–dry events.

Random forest analysis revealed that EC, TC, TN, TOC, AN, and αG had high explanatory rates for changes in the soil microbial community diversity and structure (Figure 5A). We then correlated r– (Gammaproteobacteria and Bacteroidota) and K–strategist (Alphaproteobacteria and Acidobacteroita) bacteria and the environmental factors with high explanatory rates and found that most of the K–strategist bacteria were positively correlated with six soil environmental factors, namely EC, TC, TN, TOC, AN, and αG. However, most members of Bacteroidota and some members of Gammaproteobacteria were significantly negatively correlated with the six environmental factors (Figure 5B).

### 3.6. Changes in Soil Bacterial and Fungal Community Function under Wet–Dry Events

**Figure 6:**
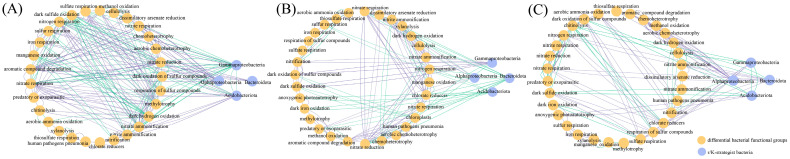
Spearman’s correlation of differential bacterial functions with r– and K–strategist bacteria of RPS (**A**), MPS (**B**), and SPS (**C**) plots in wet–dry events.

We further constructed a network of relationships between r– and K–strategist bacteria, Alphaproteobacteria and Acidobacteroita were mostly positively correlated with differentially functional groups in the RPS plots (Figure 6A), while Gammaproteobacteria were significantly negatively correlated with differentially functional groups. Alphaproteobacteria and Acidobacteroita were mostly negatively correlated with differentially functional groups in MPS plots (Figure 6B), and Alphaproteobacteria were mostly positively correlated with differentially functional groups in SPS plots (Figure 6C).

**Figure 7:**
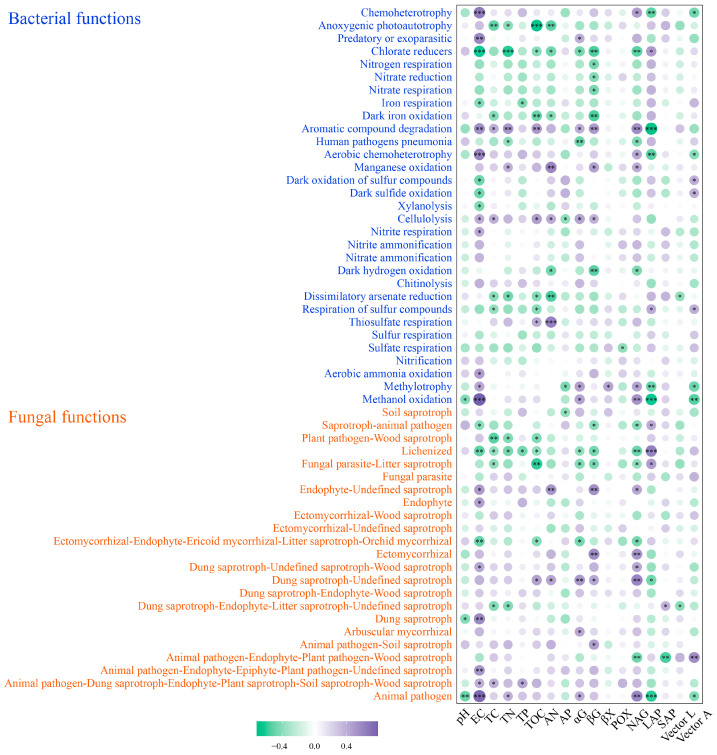
Spearman’s correlation of differential bacterial functions and differential fungal functions with physicochemical properties and extracellular enzyme activities of RPS, MPS, and SPS plots in wet–dry events. (* *p* < 0.05, ** *p* < 0.01, *** *p* < 0.001).

Under wet–dry events, pH was negatively correlated with methanol oxidation and differential bacterial function, as well as with dung saprotroph and animal pathogen with differential fungal function. Soil EC was positively correlated with most of the differential bacterial and fungal functions, whereas TOC was negatively correlated with most of the differential bacterial and fungal functions. βG was negatively correlated with the chlorate reducers, nitrogen respiration, nitrate reduction, nitrate respiration, dark iron oxidation, and dark hydrogen oxidation of differential bacterial functions, and negatively correlated with saprotroph–animal pathogen, fungal parasite–litter saprotroph, and lichenized fungal functions. βG was positively correlated with aromatic compound degradation, manganese oxidation, and the cellulolysis of differential bacterial functions, and positively correlated with endophyte–undefined saprotroph, ectomycorrhizal, dung saprotroph–undefined saprotroph, and animal pathogen–soil saprotroph with differential fungal functions. NAG was positively correlated with most of the differential bacterial and fungal functions (*p* < 0.05) (Figure 7).

## 4. Discussion

### 4.1. Large Differences in Soil Physicochemical Properties and Enzyme Activities under Wet–Dry Events

The intensity of the transition from wet to dry soil generally leads to changes in pH [46]. However, in our three sample plots, although pH differed, there was no significant difference. Perhaps the reason for the insignificant pH change in our study was that *Z. mays* and *G. max* cultivation requires water availability, despite being in a dryland agroecosystem [47]. Moreover, there were significant differences in soil EC among the three sample plots because the study area is located in the Yellow River beach area of arid and semi–arid regions, where soil salinization is severe, and sufficient water in the *O. sativa*–growing fields indirectly reduces the enrichment of soluble cations in the soil, thus decreasing the soil EC. The TC content of the soil decreased and then increased with the *O. sativa*–*Z. mays*–*G. max* rotation, showing significant differences between the sample plots. Wet soils showed increased soil carbon mineralization and TC content, which explains the higher TC content of the MPS sample plot than that of the RPS sample plot in this study [46]. The higher TOC content in the SPS sample plot was probably due to the “legacy effect” of the accumulation of plant litter and root exudates after two years of *O. sativa* cultivation [48]. 

The changes in TN content showed a pattern similar to that of TC. *O. sativa* cultivation is the main source of N_2_O, especially in wet–dry events. However, the increase in soil organic carbon mineralization through the wet–dry farming of soil could enhance the accumulation of nitrogen [49,50]. Furthermore, the increased soil nitrogen in the *G. max* field was not only caused by the increased TOC content but also by the biological nitrogen fixation ability of legumes on soil TN and soil nitrogen availability [51]. It has been demonstrated that soils with high TC content have increased soil carbon and nitrogen availability, which further confirms our conclusion that the SPS sample soil had higher TOC and AN content [52]. Furthermore, there were no significant differences in the soil TP and AP among the different sample plots in this study. In general, the higher nutrient input of soils in high–fertility fields results in a higher phosphorus content [53]. This study also speculated that crops may have higher yield and phosphorus utilization efficiency under the *O. sativa*–*Z. mays*–*G. max* rotation practice, resulting in insignificant changes in soil TP and AP content [53]. Another explanation is that the accumulation of phosphorus in the soil was a long–term land utilization, while our study was conducted on a short–term annual land rotation [54].

Numerous studies have concluded that high TC content positively affects soil extracellular enzyme activity in addition to increasing soil carbon and nitrogen availability [52,55]. In this study, soil wet–dry events also had a profound effect on soil extracellular enzyme activity, especially αG, βG, βX, NAG, and LAP. Previous studies have concluded that crop rotation affects soil extracellular enzyme activity to varying degrees [56]. In addition, the increase in soil αG and βG activities also significantly increased TOC turnover [57]. The increase in soil nitrogen also increased the activity of αG and βG, which explains the higher αG, βG, and SAP activity in the SPS sample plot in our study. Notably, in one study, soil LAP and SAP activities showed a significant negative correlation with TC and TN [58], and this study further confirmed the above conclusions. In summary, these results revealed the different potential mechanisms of hydrolase and oxidase activities in response to land–use changes.

### 4.2. Wet–Dry Events Drove the Transition of Soil r–Strategist Bacteria to K–Strategist Bacteria

Land–use changes and wet–dry events not only affect soil nutrient availability but also affect microbial community diversity [59]. In our three sample plots, the differences between soil bacterial number, diversity, and richness were not significant, but soil fungi were more sensitive to wet–dry events. Fungal communities in soil are known to respond differently to plants than bacteria because different plant species determine the diversity and richness of fungi [60]. Microbiologists divide bacterial communities into r– and K–strategists to compare the ecological characteristics and functions of different bacterial groups [19,21]. In this study, the relative abundance of r– and K–strategist bacteria was different in the three sample plots, and many studies have shown that the selection of soil microbial survival strategies is influenced by soil physicochemical characteristics, and changes in EC, TOC, and AN have a positive effect on the selection of soil bacterial survival strategies during land–use conversion [60,61]. 

Recent studies have found that, as soil organic carbon and nitrogen stocks increase, they increase the proportion of K–strategists and decrease the proportion of r–strategists [62]. Our findings were similar in that the accumulation of soil TOC and AN content with wet–dry conversion decreased the relative abundance of Bacteroidota and, at the same time, increased the relative abundance of K–strategists (Alphaproteobacteria and Acidobacteria). In addition, Luo et al. (2023) noted that increases in extracellular enzyme activities (αG, NAG, and SAP) also respond to changes in microbial taxa [62]. In this study, the enhanced correlation between Alphaproteobacteria and NAG, as well as Acidobacteria and SAP, further confirms the above conclusions. Overall, the effects of crop–planting differences on soil physical and chemical properties had positive effects on r– and K–strategist bacteria.

Reciprocal and competitive relationships among soil microorganisms can also be characterized using co–occurrence networks [30]. In our study, the network relationships between r–strategist bacteria and fungi differed between the three sample plots. *Z. mays* cultivation increased the connectivity and complexity of the network (with more nodes and edges), whereas nutrient cycling under wet–dry events changed the interaction and intensity of r–strategist bacteria and fungi, thus affecting the network relationships [63]. In addition, plant type differences are also important in shaping bacterial–fungal inter–relationships, as confirmed by many previous studies [64]. Although the network relationships in the MRS sample plots in our study were more complex, the SPS sample plots had more stable network relationships such as higher positive–to–negative ratios of edges and a larger number of modules. Reciprocal and competitive relationships among microbial taxa alter biological interactions and ecological niche sharing [65,66]. In contrast, the r–strategist bacteria (Bacteroidota) had a lower niche proportion in the SPS sample plots in the co–occurrence network, which might be due to the collaborative nutrient competition (TOC or AN) among microbial species [67].

### 4.3. Soil Environment Is an Important Affecting Microbial Function Changes under Wet–Dry Events

The impact of agricultural activities on nutrient accumulation in soil ecosystems shapes soil microbial communities and alters their functions [11,68]. In this study, bacterial and fungal functions significantly differed between the three sample plots. It has been proven that changes in the soil microbial community structure positively affect microbial functions [69], especially based on the element cycle (carbon, nitrogen, and phosphorus) [70,71]. In soil bacterial communities, most of the differential functional groups are related to the nitrogen cycle, and previous studies have found that nitrogen transformation–denitrification and nitrification directly affect nitrate concentration in wet–dry ecosystems [72]. Our research shows that an imbalance in the microbial functional response may be the dominant factor contributing to the transformation of the nitrogen cycle in RPS–to–SPS plots. Several studies have suggested that the transformation of soil wet–dry events in agroecosystems is a major factor influencing microbial properties and soil nitrogen cycling [68,73]. In particular, changes in the relative abundance of members of Gammaproteobacteria and Bacteroidota are important for explaining changes in the soil nitrogen cycle [73]. Our further analyses not only confirmed this conclusion but also revealed that bacterial taxa associated with the carbon cycle also reflected changes in soil ecological functions during wet–dry events. For example, the increase in chitinolysis and cellulolysis taxa is associated with carbon cycling under wet–dry events, and a significant correlation was observed between these taxa with EC, TOC, and AN. Then, the strength of the relationship between soil microbial habitat specificity and function provides more evidence of the co–occurring relationship between soil physicochemical properties and soil microbial communities [73].

In view of the ecological indicator role of saprotroph in land–use conversion [31], a significant correlation was observed between saprotroph groups (plant pathogen–wood saprotroph, fungal parasite–litter saprotroph, and dung saprotroph–undefined saprotroph) and TOC in our study. This research further confirmed the influence of soil physicochemical properties on the microbial community and functions under wet–dry events. Notably, the ectomycorrhizal fungi showed a strong positive correlation with βG and NAG. Soil microorganisms decompose soil organic matter and regulate soil nutrient cycling by producing various extracellular enzymes, and ectomycorrhizal fungi play an important role in plant nutrient acquisition [74]. Our study further showed the positive significance of crop rotation planting under wet–dry events for soil nutrient improvement.

## 5. Conclusions

In conclusion, the results of this study showed that different plant cultivations in a paddy–dryland rotation system affected soil physicochemical properties and enzyme activity. Moreover, the *G. max* field cultivation resulted in higher TC, TN, TOC, and AN content while also significantly increasing αG, βG, and SAP activity in the soil. In addition, crop rotation altered the r/K–strategist bacteria, and the soil environment was the main factor affecting the community structure of r/K–strategist bacteria. The co–occurrence network revealed the inter–relationship between r/K–strategist bacteria and fungi, and with the succession of land rotation, the *G. max* sample plot exhibited more stable network relationships. Random forest analysis further indicated the importance of EC, TC, TN, TOC, AN, and αG in the composition of soil microbial communities under wet–dry events and revealed significant correlations with r/K–strategist bacteria. Based on functional predictions of microorganisms, wet–dry conversion altered the functions of bacteria and fungi and led to a more significant correlation between soil nutrient cycling microbial taxa and environmental changes. This study contributes to a deeper understanding of microbial functional groups; however, this is only limited to the Yellow River beach area in northwestern China, and it is hoped that future studies can be carried out on a larger scale to better understand the potential functions of soil microbial functional groups in soil ecosystems.

## Figures and Tables

**Table 1:** Soil physicochemical properties and extracellular enzyme activities of RPS, MPS, and SPS plots in wet–dry events.

Soil Physicochemical Properties and Extracellular Enzyme Activities	RPS	MPS	SPS
pH	8.11 ± 0.04a	8.06 ± 0.04a	7.04 ± 0.02a
EC (us/cm)	815.67 ± 92.03b	1554.00 ± 159.51a	1378.67 ± 125.23a
TC (g/kg)	25.36 ± 0.55b	24.79 ± 0.86b	28.92 ± 0.67a
TN (g/kg)	0.88 ± 0.05b	0.84 ± 0.05b	1.17 ± 0.06a
TP (g/kg)	0.71 ± 0.02a	0.74 ± 0.02a	0.76 ± 0.04a
TOC (g/kg)	17.74 ± 0.82b	18.01 ± 0.99b	23.56 ± 0.65a
AN (mg/kg)	55.16 ± 2.24b	48.64 ± 1.93b	68.03 ± 2.57a
AP (mg/kg)	24.82 ± 0.70a	22.38 ± 0.54a	23.96 ± 1.24a
αG (ng/L)	42.92 ± 1.09b	45.43 ± 0.71a	47.64 ± 0.71a
βG (IU/L)	47.21 ± 1.23b	47.49 ± 0.83b	51.73 ± 0.63a
βX (IU/L)	225.21 ± 4.37a	224.73 ± 4.27a	221.70 ± 5.86a
POX (IU/L)	242.67 ± 5.59a	249.11 ± 5.77a	247.10 ± 7.11a
NAG (U/L)	40.53 ± 0.78b	42.93 ± 0.60a	44.50 ± 0.79a
LAP (U/L)	253.65 ± 4.36a	244.67 ± 5.70a	227.26 ± 5.05b
SAP (U/L)	787.96 ± 21.09a	853.59 ± 16.18a	781.08 ± 19.46a
Vector L	1.83 ± 0.009ab	1.82 ± 0.006b	1.84 ± 0.007a
Vector A	52.48 ± 0.12a	51.88 ± 0.16b	52.14 ± 0.19ab

RPS: *Oryza sativa*–planting soil; MPS: *Zea mays*–planting soil; SPS: *Glycine max*–planting soil. The differences between different sample plots are shown with lowercase letters (Tukey’s test; *p* < 0.05).

**Table 2:** Network topological features of r–strategist bacteria and functional fungi of RPS, MPS, and SPS plots in wet–dry events.

Network Parameters	RPS	MPS	SPS
Nodes	800	1007	898
Edges	821	1233	865
Positive edges	572	671	489
Negative edges	249	562	376
Number of modules	264	254	309
Modularity	0.87	0.86	0.90
Average path length	7.40	9.64	10.53
Graph diameter	23.08	29.62	30.30
Clustering coefficient	0.32	0.32	0.39
Degree centralization	0.01	0.01	0.01

RPS: *Oryza sativa*–planting soil; MPS: *Zea mays*–planting soil; SPS: *Glycine max*–planting soil.

## Data Availability

The dataset analyzed in this article is available upon request to the corresponding authors.
